# A novel serum based biomarker panel has complementary ability to preclude presence of early lung cancer for low dose CT (LDCT)

**DOI:** 10.18632/oncotarget.17477

**Published:** 2017-04-27

**Authors:** Xiaobing Wang, Xiuyi Zhi, Zhaogang Yang, Haimei Tian, Yanfen Li, Mo Li, Wenya Zhao, Chao Zhang, Teng Wang, Jing Liu, Di Shen, Cuining Zheng, Dan Zhao, Sheng Yang, Jun Qi, Hongwu Xin, Alexander Stojadinovic, Itzhak Avital, L. James Lee, Jianyu Rao, Wei Zhang

**Affiliations:** ^1^ Tumor Marker Research Center, National Cancer Center/Cancer Hospital, Chinese Academy of Medical Sciences and Peking Union Medical College, Beijing, PR China; ^2^ Department of Thoracic Surgery, Xuanwu Hospital, Capital Medical University, Beijing, PR China; ^3^ NSF Nanoscale Science and Engineering Center (NSEC), The Ohio State University, Columbus, OH, USA; ^4^ Laboratory of Clinical Biochemistry, Chinese Academy of Medical Sciences and Peking Union Medical College, Beijing, PR China; ^5^ Department of Gynecological Oncology, Cancer Institute and Hospital, Chinese Academy of Medical Sciences and Peking Union Medical College, Beijing, PR China; ^6^ Department of Medicine, Cancer Institute and Hospital, Chinese Academy of Medical Sciences and Peking Union Medical College, Beijing, PR China; ^7^ The First Peoples’ Hospital of Jingzhou City, The First Hospital and Clinical Medical School of Yangtze University, Jingzhou, PR China; ^8^ Laboratory of Oncology, Center for Molecular Medicine, Medical School, Yangtze University, Huber, PR China; ^9^ Uniformed Services University of the Health Sciences, Bethesda, Maryland, USA; ^10^ P8-Medicine Ltd, Sussex, DE, USA

**Keywords:** lung cancer, biomarker, early detection, MIC-1, screening

## Abstract

Low Dosage Computerized Tomography (LDCT) has been shown to improve early detection of lung cancer and mortality rates in high-risk individuals, which was, however, limited by specifically coverage for heavy smokers and high rates of false positivity. Here, we aim to investigate a novel biomarker for early detection of lung cancer, and further extend to concentrate high-risk subjects for increasing specificity and coverage of LDCT. We performed retrospective blinded evaluation of lung cancer and healthy controls in training and validation cohorts. Macrophage inhibitory cytokine 1 (MIC-1) alone and panel were assessed. Our data showed the sensitivity of MIC-1 was 72.2% and 67.1% for lung cancer diagnosis and early diagnosis respectively, at 96.6% specificity, which were significantly higher than Cyfra21-1, NSE CA125, CEA and SCC. At 90% specificity, the panel of MIC-1, Cyfra21-1, CA125 and CEA provided 89.5% sensitivity for early diagnosis of lung cancer, which could be used to concentrate the high-risk subjects for further LDCT screening. We conclude that MIC-1 have great capacity in early lung cancer diagnosis. The algorithmic panel of MIC-1, Cyfra21-1, CA125 and CEA could be used to refine the preselection criteria of high-risk subjects, and thus might facilitate the widespread implementation of LDCT screening.

## INTRODUCTION

Lung cancer is one of the most common human malignant tumors worldwide with considerable attendant societal costs [1, 2]. Early detection and treatment of lung cancer stands for an urgent global healthcare need and a formidable challenge in the control of this complex and deadly disease [3]. The National Lung Screening Trial concluded specificity of 73.4% and sensitivity of 93.8% for annual low-dose CT screening in high-risk smoker [4]. However, this type of scan is accompanied by high false-positive rates and limited applicable coverage. In addition, cost, concerns regarding overdiagnosis, and cumulative radiation exposure remain points of concern. Affordable noninvasive testing such as blood-based biomarkers could potentially improve the positive predictive value of precise LDCT screening, and more importantly, concentrate the potential risk subjects which may in turn extend the applicable coverage for screening potential subjects, such as the non-smoking potential risk subjects.

Although recent advances in molecular diagnostics have generated many candidate biomarkers for lung cancer, the seromarkers developed thus far have not been recognized as ideal biomarkers due to the limited sensitivity and specificity either singularly or as a panel of markers [5, 6]. The traditional lung cancer biomarkers remain the most studied biomarkers for lung cancer, especially in developing countries [7], which show an increased rate of positivity as the cancer stage advances, but could hardly be serve as standalone indicators of disease at its earliest stages. For example, the most sensitive Cyfra21-1 alone had a reported sensitivity threshold of 44% [8, 9], and the most sensitive early diagnosis of lung cancer methodology with a biomarker panel remains to be determined, though preliminary analysis suggest a relevant sensitivity threshold of > 60% [10, 11]. Therefore, new serologic biomarkers with sufficient sensitivity to reliably diagnose asymptomatic patients with lung cancer should be investigated.

Growing evidence indicates that abnormal immune response is involved in cancer patients before clinical confirmation of disease [12, 13]. Therefore, investigating the associated cytokines involved with the immune response to the developing cancer may be a promising approach to identify biomarkers that can detect cancer at an early stage [14]. Macrophage inhibitory cytokine 1 (MIC-1/GDF15) was originally discovered in macrophage cells and associated with immune inhibition [15–17]. Serum MIC-1 is substantially increased in disease states caused by inflammation and invasive malignancy [18–21]. Numerous studies have demonstrated that MIC-1 plays a valuable function in the biology of carcinogenesis [22–24]. This study aims to explore the value of MIC-1 as a biomarker in the onset of lung cancer, and furthermore, using MIC-1 as a critical factor, construct predictive panel for the preselection of potential risk subjects, which may facilitate the widespread implementation of LDCT screening and enhance its cost-effectiveness.

## RESULTS

### Serum MIC-1 is elevated earlier and more progressively than any available clinical biomarkers in lung cancer

**Figure 1:**
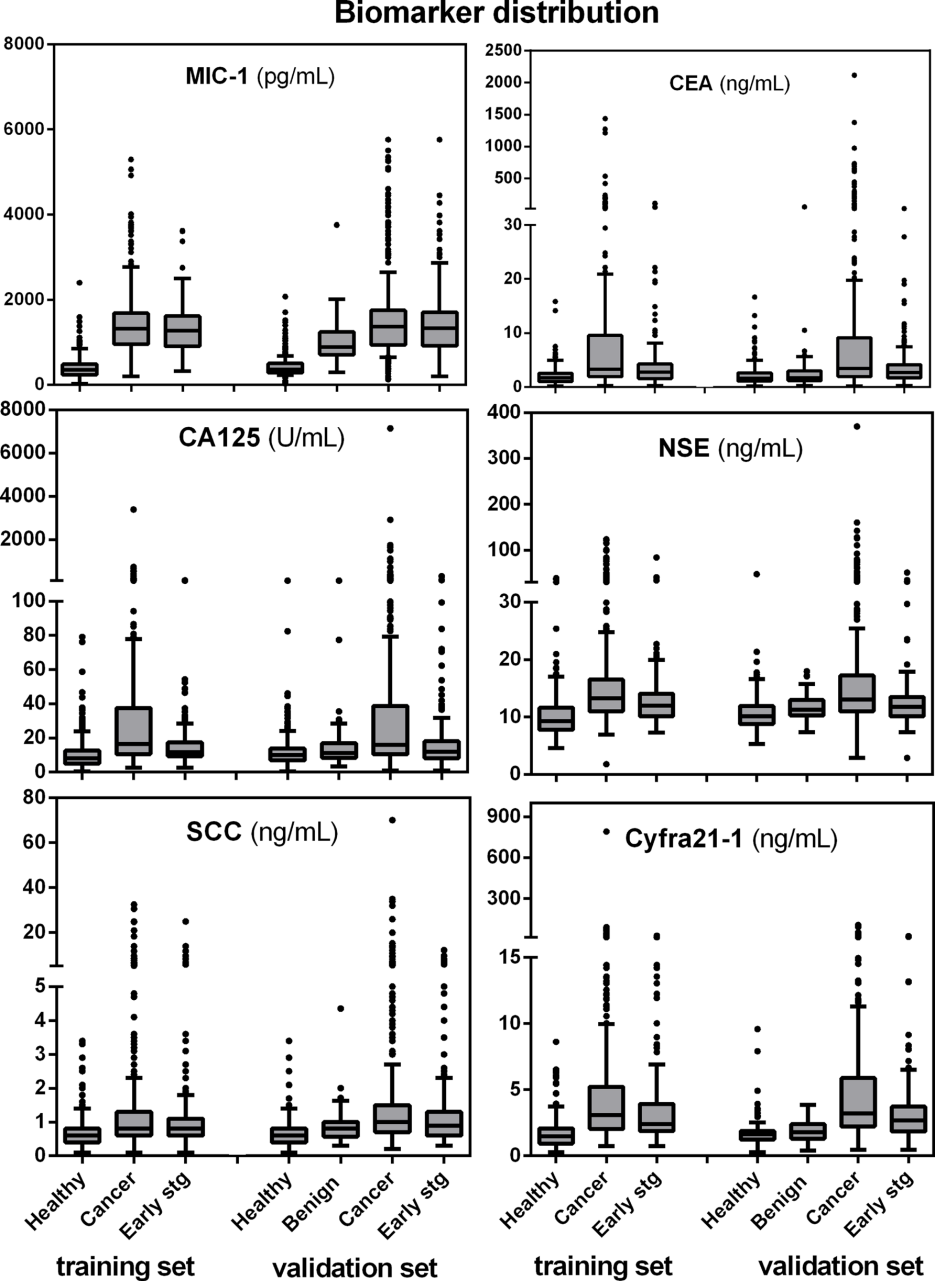
Boxplot display of biomarker distributions for controls, lung cancer cases, the subset of early stage (I and II) cases and benign conditions, by training and validation set assignment Horizontal box boundaries and midline denote sample quartiles. Whiskers mark *adjacent values: upper adjacent value = largest marker value x such that x < = 75th percentile + 1.5 * interquartile distance. Similarly, lower adjacent value = lowest marker value such that x > = 25th percentile -1.5 * interquartile distance. The interquartile distance (IQR) = 75th–25th percentile.

First, we assessed the serum levels of MIC-1 in training group. MIC-1 level was significantly increased in lung cancer patients compared to healthy controls (*p* < 0.001; Figure 1 and Supplementary Table 1). Notably, high levels of MIC-1 was observed in early-stage (Stage I/II) lung cancer patients (*p* < 0.001). Moreover, when all patients with lung cancer were grouped according to TNM classification, the gradual increasement in serum MIC-1 levels was evident (*P* = 0.042), with higher levels in advanced patients compared with early-stage patients (Table 1), implying the positive correlation of MIC-1 with lung cancer progression. Further analysis showed that the level of serum MIC-1 was significantly higher in low grade lung cancer, distant metastasis than in high grade tumors or in the absence of distant metastasis, respectively (Table 1).

**Table 1:** Correlation between MIC1 serum level and clinicopathological variables of lung cancer

	Training set							Validation set						
Variables	*N*	min	max	Median	Mean	IQR	*P*	*N*	min	max	Median	Mean	IQR	*P*
TNM stage														
stage I	95	319.3	3618.0	1274.5	1348.3	766.3	0.042	123	195.2	5759.0	1245.4	1359.6	757.2	0.017
stage II	60	347.8	2465.9	1222.0	1249.5	731.5		76	397.5	4272.7	1355.0	1556.1	940.1	
stage III	100	396.6	5060.1	1365.9	1551.8	792.2		112	269.2	4493.4	1317.2	1454.6	810.0	
stage IV	95	196.6	5292.4	1384.9	1600.4	644.0		100	126.0	5506.1	1477.8	1849.6	852.7	
T classification														
T_1-2_	226	317.3	5292.4	1272.3	1342.2	686.3	0.0004	279	195.2	5759.0	1324.5	1493.2	787.6	0.35
T_3-4_	116	196.6	5060.1	1470.0	1685.1	788.3		120	126.0	5277.8	1405.5	1580.2	833.2	
Unkown	8							12						
N classification														
N_0_	115	319.3	3770.2	1294.9	1395.0	760.3	0.87	161	195.2	5759.0	1245.4	1380.0	743.2	0.182
N_1-3_	224	196.6	5292.4	1326.1	1471.7	726.3		233	126.0	5506.1	1384.0	1590.6	838.1	
Unkown	11							18						
Remote metastasis														
M_0_	255	319.3	5060.1	1276.2	1405.1	742.1	0.039	311	126.0	5759.0	1315.9	1430.7	788.1	0.0006
M_1_	95	196.6	5292.4	1395.6	1599.7	681.7		100	151.7	5506.1	1488.5	1884.2	858.8	
Differentiation														
Poorly	140	196.6	5292.4	1347.4	1522.7	713.1	0.072	181	126.0	5506.1	1465.0	1660.1	830.1	0.0001
Moderately	169	380.9	3773.7	1336.3	1439.6	694.8		161	269.2	5354.4	1310.9	1532.3	775.2	
Well	41	352.5	5060.1	1006.9	1312.6	671.6		69	195.2	5759.1	1092.8	1249.3	800.6	
Pathological type														
SCLC	31	468.5	3680.0	1338.5	1500.4	725.7	0.953	40	126.0	4450.7	1401.7	1603.6	749.6	0.665
NSCLC	319	196.6	5292.4	1319.0	1453.8	731.7		371	151.7	5759.0	1365.6	1534.3	825.5	
Adenocarcinoma	114	319.3	3773.7	1381.3	1471.8	723.3	0.182	114	151.7	4050.6	1369.7	1413.6	714.6	0.756
Squamous cell carcinomas	197	196.6	5292.4	1276.6	1441.7	725.4		247	195.2	5759.0	1347.8	1579.63	863.9	
others	8							10						

We also compared MIC-1 with other previously investigated lung cancer serum biomarkers. Although serum from patients with lung cancer showed elevated levels of Cyfra21-1, NSE, CA125, CEA and SCC compared with the healthy group (Supplementary Table 1), our data showed that as single markers, MIC-1 was found to be elevated much earlier stage in the course of the disease and more progressively than any other five clinically available biomarkers. The distributions of all six biomarkers in lung cancer (early-stage lung cancers listed separately), and healthy controls were showed and compared using boxplots (Figure 1). The result from the validation group confirmed these findings, and also showed that serum MIC-1 levels represented a stepwise increasement in benign diseases and lung cancers in comparison with healthy controls, indicating that the overexpression of MIC-1 started likely at the development of lung cancer (Figure 1; Table 1).

### Serum MIC-1 significantly improves lung cancer diagnosis, especially early stage diagnosis

**Figure 2:**
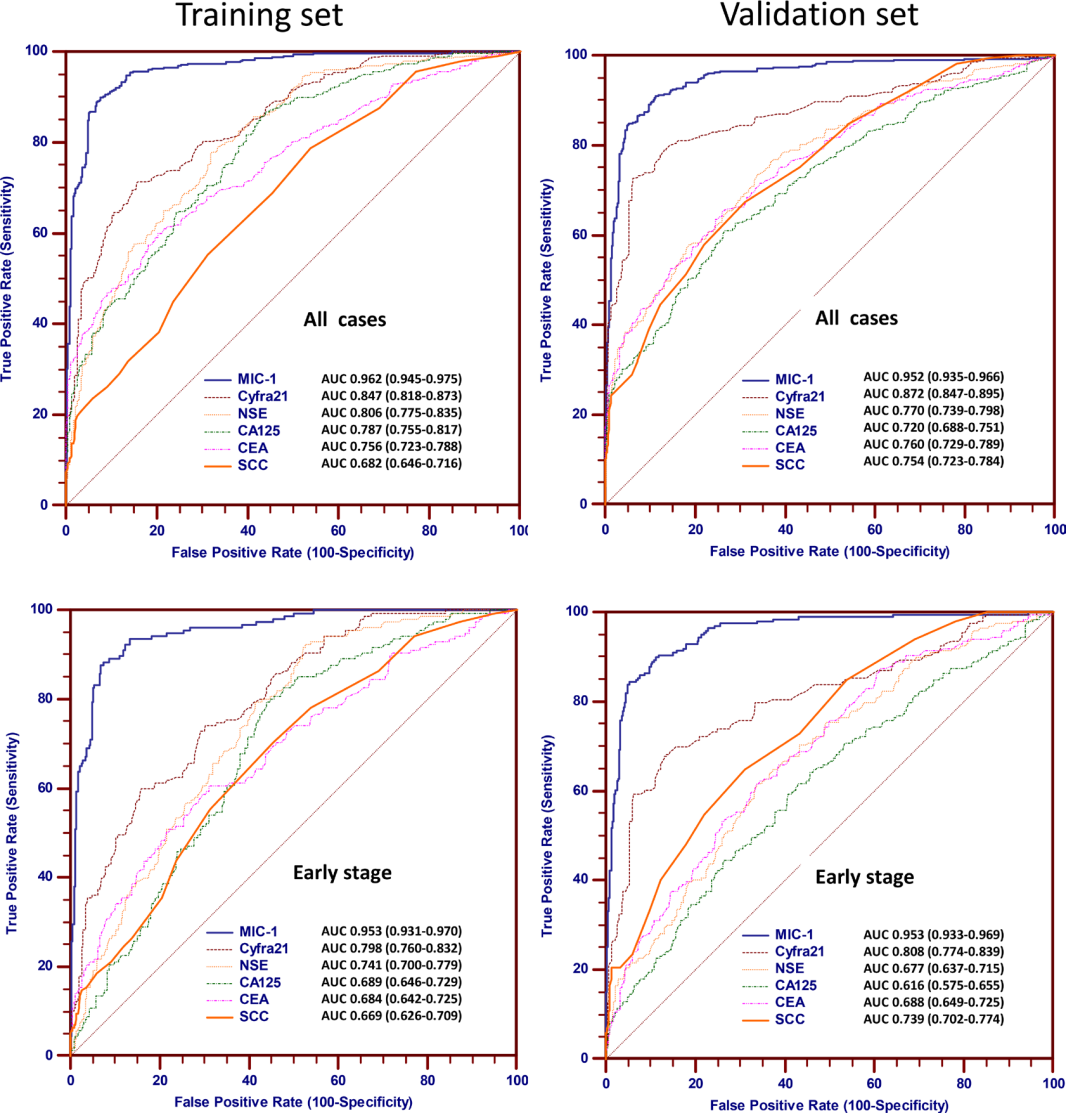
Biomarker ROC curves for cases and controls assigned to the training group and validation group ROC curves of MIC-1, Cyfra21-1, NSE, CA125, CEA and SCC for discriminating between lung cancer and control in the training group (upper left) and validation group (upper right). ROC curves of MIC-1, Cyfra21-1, NSE, CA125, CEA and SCC for discriminating between early-stage lung cancer and control in the training group (lower left) and validation group (lower right).

Next, we generated receiver operating characteristic curves (ROC) to evaluate the utilization of the serum MIC-1 protein as a non-invasive diagnostic marker for lung cancer. In training group, the obtained ROC curve of MIC-1, Cyfra21-1, NSE, CA125, CEA and SCC for lung cancer and early-stage lung cancer are graphically shown in Figure 2. MIC-1 had the greatest ability to distinguish lung cancer cases from healthy subjects. The area under ROC curve (AUC) of MIC-1 was 0.962 for all cases, whereas the AUCs of Cyfra21-1, NSE, CA125, CEA and SCC were 0.847, 0.806, 0.787, 0.756 and 0.682, respectively. MIC-1 had significant discriminatory value, especially when only early-stage (Stage I/II) lung cancer samples were tested. The AUC of MIC-1 was 0.953 for early-stage cases, whereas the AUCs of Cyfra21-1, NSE, CA125, CEA and SCC were 0.798, 0.741, 0.689, 0.684 and 0.669, respectively. These data suggest that serum MIC-1 can significantly improve lung cancer diagnosis, especially early diagnosis.

We further calculated the detection sensitivity of MIC-1 for all lung cancer and early stage I/II lung cancer at various specificity cut-offs (Supplementary Table 2). For facilitating clinical application, we set 1,000 pg/ml as the cutoff value for warranting acceptable specificity. The sensitivity of MIC-1 were 72.2% and significantly higher than that of Cyfra21-1 (46.6%), NSE (20.0%), CA125 (26.6%), CEA (37.7%) and SCC (21.1%), with comparable specificity (Supplementary Table 3). Notably, the sensitivity was 67.1% in detecting early-stage lung cancer, which was significantly higher than that of Cyfra21-1 (34.2%), NSE (6.5%), CA125 (5.8%), CEA (20.6%) and SCC (15.5%). These data further show that serum MIC-1 could be used for lung cancer detection, especially early lung cancer.

To validate the findings above, we performed similar ROC analysis in the validation group with the healthy individuals as the control group. The results obtained with the training and the validation groups were in good agreement (Figure 2 and Supplementary Table 3). On the other hand, as shown in Supplementary Table 2, when patients with benign conditions were used as the control population in the validation group, the sensitivity of MIC-1 for detecting early stage lung cancer was 65.3%, which is significantly superior to Cyfra21-1 (31.2%), NSE (3.5%), CA125 (9.5%), CEA (17.6%) and SCC (20.6%). We further performed ROC curve analysis in subgroups of patients, stratified by histology against the control healthy group. A summary of the AUCs for each of the subgroups is shown in Supplementary Table 4, showing that the associated AUCs are generally similar across all subgroups. Thus, we concluded that MIC-1 has similar discriminating ability for all of the major histological subtypes of lung cancer.

### The four biomarker panel MIC-1, Cyfra21-1, CA125 and CEA improves lung cancer diagnosis, especially early diagnosis, more than MIC-1 alone

**Figure 3:**
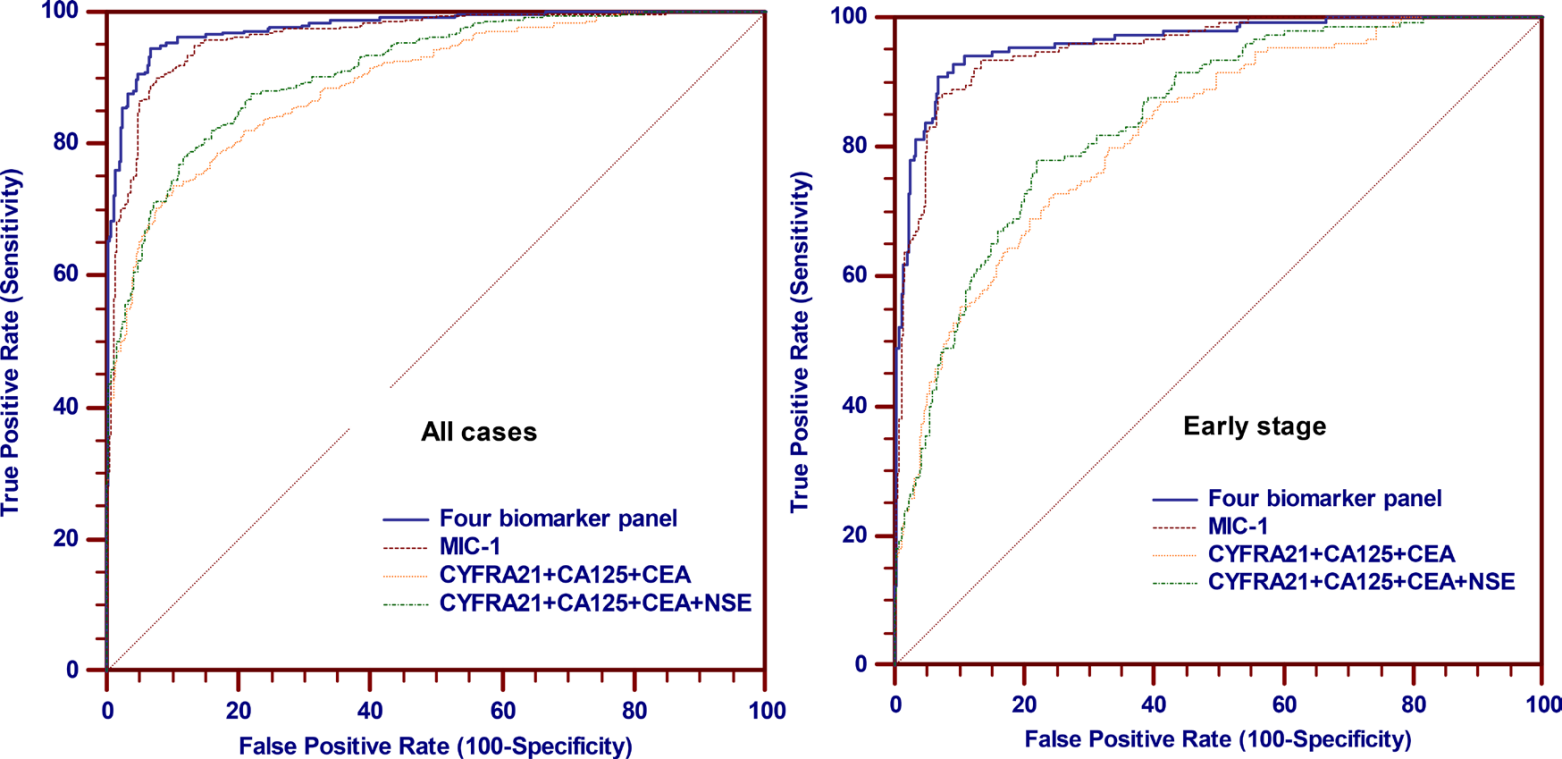
Performance of MIC-1 combining with other biomarkers in the detection of lung cancer in the training group (Left) ROC curves of four biomarker panel, MIC-1 alone, Cyfra21-1, CA125, CEA combination and Cyfra21-1, CA125, CEA, NSE combination for all lung cancer cases (*N* = 350) and controls (*N* = 350); (Right) ROC curves of four biomarker panel, MIC-1 alone, Cyfra21-1, CA125, CEA combination and Cyfra21-1, CA125, CEA, NSE combination for early-stage lung cancer cases (*N* = 155) and controls (*N* = 350).

To further improve diagnostic sensitivity, we used logistic regression on raw values of each marker to establish a model with the above-mentioned serological biomarkers. Linear regression p-values were calculated to evaluate whether a single marker of the panel significantly increases the differences between cases and controls, which was described in Supplementary Table 5. A four-biomarker panel consisting of MIC-1, Cyfra21-1, CA125 and CEA demonstrated superior performance compared with other combination (Supplementary Table 6). Therefore, we fit an algorithm model using results from the training groups: Logit [probability of lung cancer] = −7.7157 + 0.005752* MIC-1 + 0.6275*Cyfra21-1 + 0.03770*CA125 + 0.1101*CEA. In the ROC analysis, the four-biomarker panel consisting of MIC-1, Cyfra21-1, CA125 and CEA provided a significant improvement in both sensitivity and specificity. At a cut-off probability value of 0.559, this four-biomarker panel provided 90.6% sensitivity (95% CI, 87.0 to 93.4%) for lung cancer at 95% specificity (Figure 3). Furthermore, the panel of four-biomarker represented comparable classification for the three most common histologic types of lung cancer: Adenocarcinomas (sensitivity, 90.4%), squamous cell carcinomas (sensitivity, 92.1%) and SCLC (sensitivity, 83.9%). With this unique four-biomarker panel, we were also able to detect both NSCLC and SCLC tumors.

In the analysis of patients with early-stage lung cancer, the four-biomarker panel including MIC-1 identified a sensitivity of 83.9% at a specificity of 95%; notably, the sensitivity for early stage lung cancer by the four-biomarker panel would increase up to 92.9% (95% CI, 87.7% to 96.4%) when the specificity was decreased to 90%, at a cut-off probability value of 0.267.

### The significant improvement of lung cancer diagnosis, especially early diagnosis, by the four biomarker panel is validated

**Figure 4:**
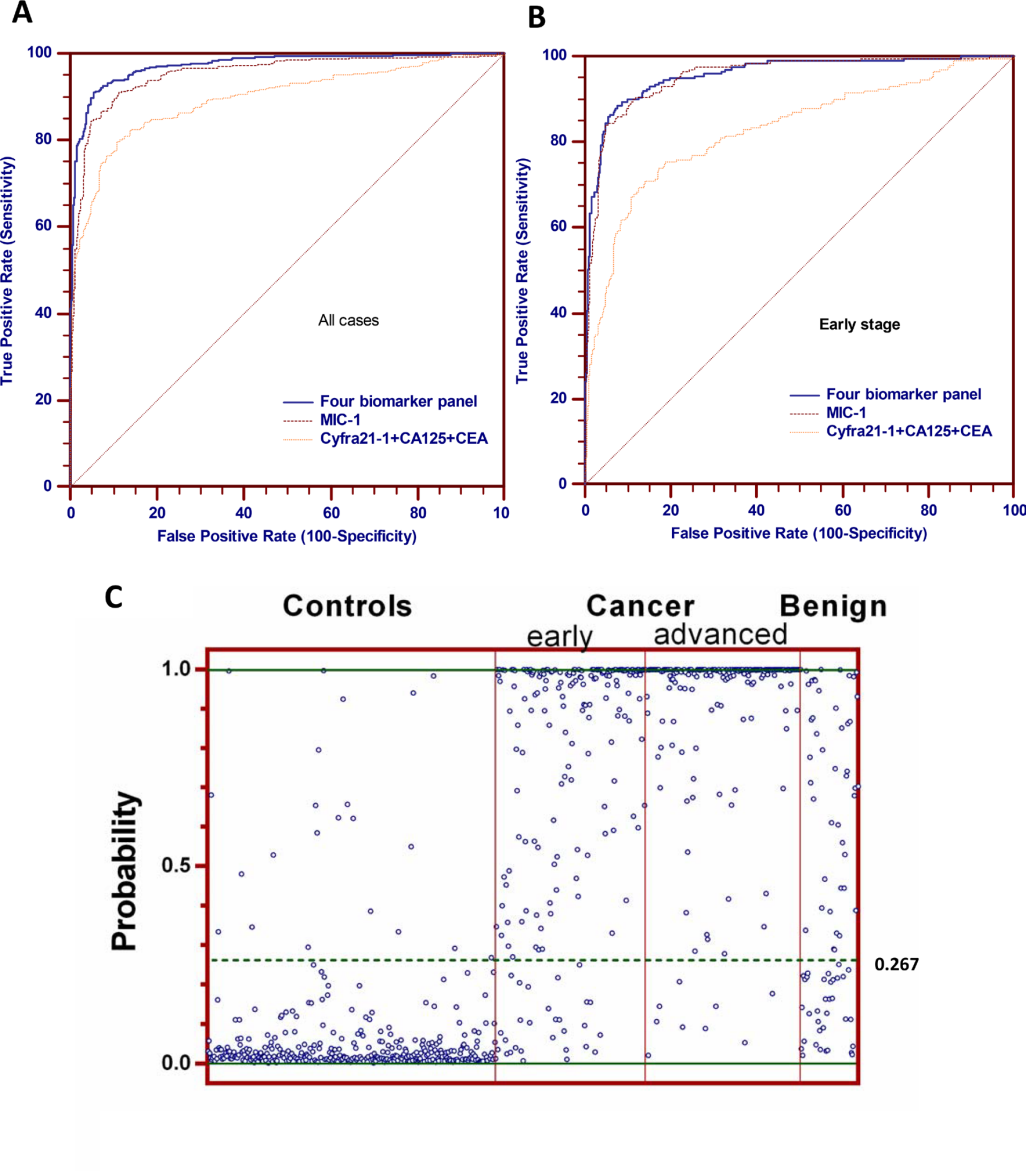
Performance of the four biomarkers panel (MIC-1, Cyfra21-1, CA125 and CEA) in the validation group (**A**) ROC curves of four biomarker panel, MIC-1 alone and Cyfra21-1, CA125, CEA combination for all lung cancer cases in the validation group. (**B**) ROC curves of four biomarker panel, MIC-1 alone and Cyfra21-1, CA125, CEA combination for early-stage lung cancer cases in the validation group. (**C**) scatterplot of the model predicted disease probabilities by disease group for subjects in the validation group.

We then analyzed the performance of the predictive model in the validation group, to determine whether the four-biomarker panel could differentiate normal from lung cancer samples in the independent group. The panel of four-biomarker conducted well for characterization of patients with lung cancer, offering 0.974 AUC and 90.3% sensitivity (95% CI, 87.0% to 93.0%) at 95% specificity, which was both significantly higher than MIC-1 alone and the other three biomarker combination tested (Figure 4A). In the analysis of patients with early-stage lung cancer and the healthy subjects used as the control, the panel showed 0.957 AUC, 84.4% sensitivity (95% CI, 78.6% to 89.2%) at 95% specificity, and 89.5% sensitivity (95% CI, 84.3% to 93.3%) at 90% specificity, which is comparable to the 92.6% sensitivity at 90% specificity from the training group (Figure 4B). The result from validation group testing further confirmed and demonstrated the superior performance of the four-biomarker panel for lung cancer, especially for early-stage lung cancer.

The performance of the final predictive model for early-stage lung cancer was evaluated using the previously identified cutoff value 0.267 for 90% specificity of the training group. The sensitivity and specificity of the four-biomarker panel was 88.4% and 93.1%, respectively. For 78 samples obtained from patients diagnosed with benign conditions, this four-biomarker panel misclassified 56.4% blinded benign samples as cancer, more specifically, 52.1% benign tumor, 57.9% lung inflammation and 54.5% pulmonary tuberculosis samples were misclassified as cancer (Figure 4C). The four-biomarker panel provide inferior sensitivity results in the validation group (88.4%) compared with the training group (92.6%), whereas the specificity increased from 90% to 93.1%, indicating that there may be some inherent population-based differences, and consequently, the cutoff of probability for 90% specificity could be optimized. This difference could be further refined in planned large scale multicenter studies.

## DISCUSSION

Lung cancer is well-known as a ‘silent killer’. Early signs and symptoms, if any, are indistinct and non-specific, and the majority of patients appear with advanced disease. Advanced lung cancer prognosis is dismal, despite aggressive therapy [2, 3, 25]. However, there is currently no good candidate marker for the early detection of lung cancer. In this study, we evaluated MIC-1 as a candidate novel seromarker for the detection of early-stage lung cancers. To our knowledge, the present research is the first in-depth exploration into the potential clinical significance of MIC-1 as a biomarker in lung cancer. We analyzed serum MIC-1 in patients with lung cancer, and demonstrated the diagnostic significance of MIC-1 and the combination of MIC-1 with clinically available biomarkers to discriminate normal tissue from lung cancer, especially early-stage I/II lung cancer with high sensitivity without compromising specificity.

First, in the retrospective research reported here, we have discovered that serum MIC-1 was increased from the early stage and correspond with progression of lung cancer. The elevations of MIC-1 level in patients with early-stage lung cancer are not well understood, since significant levels of tumor-associated products were usually detected in serum at advanced stages of tumor development. However, the known over-expression of MIC-1 by endothelial cells and macrophages in response to inflammatory signals suggests that MIC-1 may act as an important cytokine and play a specific function in inflammatory and immune responses to tumor formation [26–28]. In addition, MIC-1 may act as paracrine/autocrine cytokine or circulating hormone to stimulate/inhibit the formation/remodeling of new blood vessels [22, 29, 30]. The growth of new blood vessels, known as angiogenesis, is an important component of tumorgenesis and further uncontrolled tumor growth and metastasis. Therefore, elevated levels of the serum MIC-1 may exert an important action in growth/progression of lung cancer. Taking these factors into consideration, MIC-1 could be a promising candidate biomarker to detect early stages of the disease through its elevation in the inflammatory microenvironment of the tumor. Further analysis on the value of MIC-1 in the development of lung cancer and early detection of early stage cases is warranted.

In our study, we also compared the distributions of MIC-1 with other biomarkers, Cyfra21-1, NSE, CEA, CA125 and SCC, which are commonly used as lung cancer biomarkers in clinic [31]. Our comparative analysis further demonstrates the diagnostic potential of MIC-1 for lung cancer, as its ROC performance was significantly better than the other five markers tested. As a single marker, MIC-1 is the most promising candidate of the six biomarkers tested, especially in early-stage disease. Consequently, the use of MIC-1 could enhance the potential of treating lung cancer in its early stages and this could translate into improved cancer outcomes. Additionally, we tested the above six serum biomarkers in an independent (validation) cohort of lung cancer patients and found that the sensitivity was slightly different. Differences in patient populations tested may explain the subtle differences observed in sensitivity within the training and validation groups. These results clearly imply that MIC-1 could be served as a much more clinically potential biomarker than the other five clinically available biomarkers for early detection of lung cancer.

Numerous results have concluded that the formation of tumor is associated with chronic inflammation [32]. Both lung cancer cells and inflammatory cells secreting MIC-1 may illustrate the higher levels of MIC-1 detected in lung cancer patients. Our data showed that MIC-1 levels in benign disease subjects were significantly higher than healthy controls; while significantly lower than lung cancer group. Considering that elevated serum MIC-1 were detected in subjects with benign diseases and the imaginable interference of the chronic inflammation, we assessed MIC-1 levels in a group of benign disease subjects to study whether MIC-1 can differentiate early-stage lung cancer and non-cancerous conditions including benign lung tumor, pulmonary tuberculosis and lung inflammation. Our ROC results showed that serum MIC-1 was insufficient to differentiate patients with early-stage lung cancer from benign lung tumors, although AUC value was found to approximate that of Cyfra21-1 and to be superior to NSE, CA125, CEA and SCC.

Although the use of MIC-1 alone indicated a promising future for the early diagnosis of lung cancer, we continued to investigate the development of biomarker panels involving this highly sensitive MIC-1, further increasing the performance of early diagnosis. With the use of a logistic regression model, we found that MIC-1 and the other three biomarkers together (MIC-1, Cyfra21-1, CA125 and CEA) are able to reliably discriminate lung cancer samples and healthy control samples. Combinations of these biomarkers could improve diagnostic sensitivity which is preferable to the sensitivity of each of the four traditional biomarkers alone, while maintaining the high diagnostic specificity. We therefore have characterized a four-marker panel (MIC-1, Cyfra21-1, CA125 and CEA) that allows classification of lung cancer with wonderful sensitivity and specificity. This multi-marker panel may act as a prototype of initial screening for asymptomatic subjects. Moreover, our data further indicate that MIC-1 is equally effective in NSCLC and SCLC, as well as with adenocarcinomas and squamous cell carcinomas. The findings were validated in an independent validation group analysis. Although further analyses of more cases are needed to verify and expand upon the present data, our results strongly suggest that the complement of MIC-1 to the current lung cancer biomarkers may greatly improve the detection sensitivity for lung cancer where these markers are currently utilized clinically.

Currently, studies for early detection are mainly concentrated on the screening of preselected high-risk subjects. These mainly composed of populations with specific genetic predisposition for the formation of lung cancer, along with epidemiological information, such as heavy smoking history [33–35]. It has been suggested that a method with sensitivity and specificity above 90% would benefit high-risk subjects [36]. Our study has demonstrated that the sensitivity of the four biomarker panel for early-stage lung cancer was 92.9% and 89.5% in training and validation group, respectively, with specificity at 90% for both settings. Therefore, screening for patients with lung cancer would likely need two “steps” to concentrate the population for implement of cost-effective screen. The first step could be epidemiological factor analysis and the second step could be this non-invasive four-biomarker panel. The panel may be used as a ‘filter’ to identify high-risk subjects, which may in turn improve the positive predictive value of precise screening tools in cost-effective way.

However, it also should be cautious that lung cancers could be revealed with a panel of MIC-1 and the three other biomarkers, while not all subjects detected with this means would suffer from lung cancer, since MIC-1 and the combination values are elevated in patients with benign diseases. Based on the condition that the cutoff was assigned to exclude healthy individuals, any subjects with positive test result were likely to be in unhealthy condition that at a minimum would need additional medical assessment. In such cases, the predictive model should be regarded as helpful for the subjects, no matter which disease was involved. In conclusion, this research presents the framework for the construction of a clinically relevant strategy that could supply a cost-effective and sensitive method to detect early stage lung cancer with the aim of reducing disease-associated mortality.

## MATERIALS AND METHODS

### Serum samples and study design

Serum from 350 lung cancer patients and 350 healthy subjects were collected as the training group from January 2008 to March 2010. Additional independent serum samples from 411 lung cancer patients, 78 benign patients (48 cases of benign tumor, 19 cases of Lung inflammation, and 11 cases of pulmonary tuberculosis) and 389 healthy subjects were collected as the validation group from January 2011 to March 2014. All samples were collected from the Cancer Hospital, Chinese Academy of Medical Sciences (CICAMS, Beijing, China). Patients with lung cancer and benign disease were confirmed by histopathological analysis (Lung puncture, bronchoscopy sampling or surgery), according to the criteria defined by the American Joint Committee on Cancer. Healthy subjects were confirmed by their negative results in X-ray, ultrasound and CT examination. The clinical characteristics of the subjects are listed in Table 2. Blood samples were obtained from all study cases involved in our present study. For all lung cancer patients, samples were obtained prior to first treatment.

**Table 2:** Clinical characteristic of the studied subjects

Histology	Training group									Validation group								
	No.of Patients	Age (years)			Sex(No.of Patients)		Grade (No.of Patients)			No.of Patients	Age (years)			Sex(No.of Patients)		Grade (No.of Patients)		
		range	median	mean	male	female	1	2	3		range	Median	mean	male	female	1	2	3
Healthy controls	350	20–80	59	57.7	162	188				389	19–78	58	57.9	205	184			
Benign lung disease										78	22–75	57	56.6	39	39			
benign tumor										48	22–75	57	56.8	23	25			
Lung inflammation										19	47–68	55	55.6	12	7			
pulmonary tuberculosis										11	26–75	57	57.5	4	7			
Lung cancer	350	27–85	60	59.6	218	132	140	169	41	411	28–87	60	59.9	267	144	181	161	69
stageI	95	27–76	61	61.1	58	37	21	47	27	123	28–87	60	59.3	71	52	29	52	42
stageII	60	40–85	59	60.3	37	33	18	39	3	76	42–82	62	51.8	51	25	31	33	12
stageIII	100	38–80	60	59.6	66	34	48	49	3	112	38–86	59	60.1	80	32	64	43	5
stageIV	95	36–78	57	57.5	57	38	53	34	8	100	35–78	59	58.7	68	32	57	33	10
NSCLC	319	27–80	60	59.6	195	124	110	168	41	371	28–87	60	59.7	235	136	141	161	69
squamous cell carcinoma	114	27–80	62	61.1	90	24	44	65	5	114	28–86	62	61.5	99	15	51	60	3
adenocarcinoma	197	33–78	59	58.9	101	96	62	100	35	247	32–87	59	58.8	128	119	84	87	76
other	8	37–69	57	56.4	4	4	4	3	1	10	38–74	62	60.3	8	2	6	4	0
SCLC	31	42–85	58	59.1	23	8	30	1	0	40	46–82	62	61.4	32	8	40	0	0

The characterization and validation of serum MIC-1 and its combination were divided into 2 study phases. In phase 1 (training) study, serum MIC-1 and other biomarkers were tested and evaluated for lung cancer in training group, and a probability algorithm was generated by logistic regression. In the phase 2 (validation) study, serum samples were evaluated independently for confirmation of diagnostic parameters and cross-validation of the probability algorithm model in the validation group (Supplementary Figure 1). The study has been approved by the Ethics Committee of CICAMS.

### Serum MIC-1 detection by in-house ELISA method

Serum MIC-1 was detected by a sensitive ELISA, which produced by CICAMS as detailed previously [37, 38]. All samples were analyzed using ELISA assays on the same day according to the “Standards for the reporting of diagnostic accuracy studies (STARD) initiative” (Supplementary Table 7). Each test included two standard control (CV < 12%). All serum samples were duplicately assayed.

### Serum CEA, CA125, NSE, SCC and Cyfra21-1 assay

We detected a panel of five biomarkers, namely CA125, CEA, NSE, Cyfra21-1and SCC. The serum levels of CEA, CA125, NSE and Cyfra21-1 were tested by electrochemiluminescence immunoassay (ECLIA) kits using Elecsys 2010 (Roche, America). The levels of SCC in the serum were detected by SCC assay kit using an ARCHITECT I2000SR immune analyze system (Abbott, America). Each test included a standard control (CV < 5%).

### Statistics

The Mann-Whitney test and Kruskal–Wallis test were performed to measure the serum MIC-1 between unpaired groups and among all groups, respectively. ROC curve was used to identify the diagnostic information. Multivariable logistic regression model was conducted to corporate diagnostic performance of biomarkers. The clinical cut-off value for MIC-1 was assigned as the mean value plus two point five standard deviations of healthy individuals, and the clinical cut-off values for CEA, CA125, NSE, Cyfra21-1 and SCC were based on the manufacturer's protocols. The statistical activity was operated with the Statistical Package for the Social Sciences, version 19.0 (SPSS). *P* value of < 0.05 for a two-sided test was considered to be statistically significant.

## SUPPLEMENTARY MATERIALS FIGURES AND TABLES





## Abbreviations

MIC-1: Macrophage inhibitory cytokine 1
LDCT: Low Dosage Computerized Tomography
ROC: Receiver operating characteristic.
