# Supplementary material for: A novel serum based biomarker panel has complementary ability to preclude presence of early lung cancer for low dose CT (LDCT)
Source: Oncotarget. 2017 Apr 27;8(28):45345–55. doi: 10.18632/oncotarget.17477 (PMC5542191; doi:10.18632/oncotarget.17477)
Supplement: Supplementary file 1 [file oncotarget-08-45345-s001.pdf]

A novel serum based biomarker panel has complementary ability to preclude presence of early lung cancer for low dose CT (LDCT)

Supplementary Materials

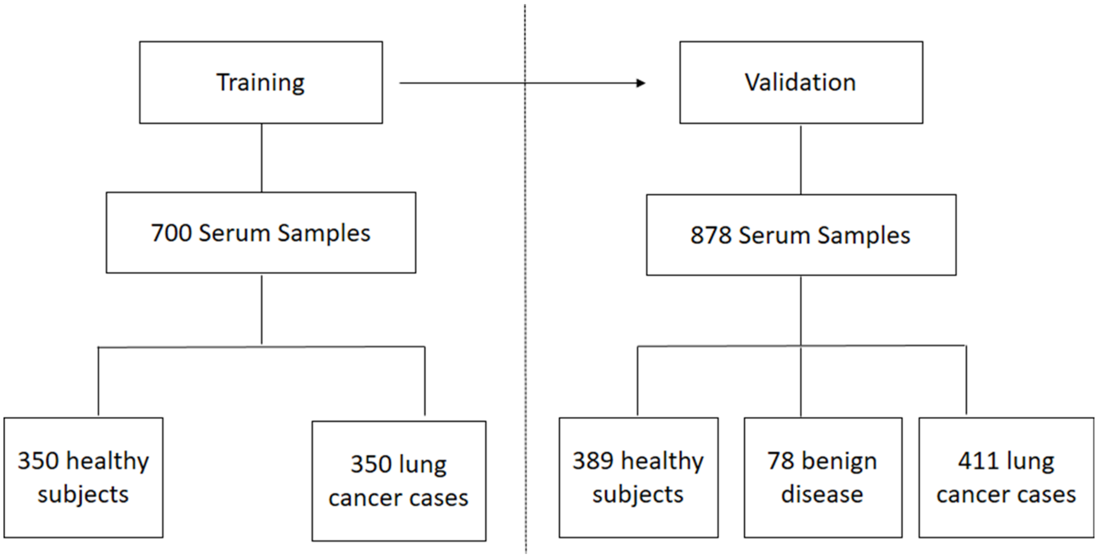

Supplementary Figure 1: The research design for the characterization and validation of serum MIC–1 and its combination in the diagnosis of Lung cancer (flow chart).

Supplementary Table 1: Statistics of each marker in healthy, benign and cancer patients of both training group and validation group

		Training group						Validation group					
Marker	Group	#	min	max	median	mean	IQR	#	min	max	median	mean	IQR
MIC-1	stage I/II	155	319.3	3618	1272.9	1310.1	712.4	199	195.2	5759	1324.5	1434.7	788
	All cases	350	196.6	5292.4	1321	1458	726.5	411	126	5759	1367.9	1541.1	815.2
	benign condition							78	290.3	3754.5	884.7	996	529.7
	healthy control	350	21	2398.9	348.2	397.2	147.4	389	23	2071.7	363.8	423.7	215.8
Cyfra21-1	stage I/II	155	0.7	27.8	2.4	3.6	2.1	199	0.4	24	2.7	3.2	1.9
	All cases	350	0.7	793	3.1	8.1	3.2	411	0.4	106.9	3.2	6	3.7
	benign condition							78	0.4	3.9	1.8	1.9	1.1
	healthy control	350	0.3	8.6	1.5	1.6	1.1	389	0.3	9.6	1.6	1.6	0.7
NSE	stage I/II	155	7.3	84.2	12	13.1	3.9	199	2.9	51.1	11.8	12.6	3.3
	All cases	350	1.8	124.1	13.3	17.8	5.6	411	2.9	370	13.1	18.5	6.2
	benign condition							78	7.3	18	11.3	11.9	2.7
	healthy control	350	4.6	39.2	9.3	10.2	3.9	389	5.3	47.9	10.2	10.6	3.2
CA125	stage I/II	155	2.7	113.6	11.8	15.8	8.1	199	1	309.5	12.1	19.6	9.9
	All cases	350	2.7	3390	16.6	50.5	27	411	1	7138	15.9	84.8	28.3
	benign condition							78	3.3	103.1	11.3	15.1	8.4
	healthy control	350	0.5	79.2	8.1	10.4	7.6	389	0.5	110.2	10	12	6.9
CEA	stage I/II	155	0.3	114.2	2.8	6.3	2.7	199	0.3	30.4	2.7	3.7	2.5
	All cases	350	0.3	1437	3.3	29.3	7.7	411	0.2	2115	3.5	38.9	7.2
	benign condition							78	0.3	58.2	1.7	2.9	1.8
	healthy control	350	0.2	15.9	1.7	2	1.6	389	0.2	16.7	1.7	2.1	1.5
SCC	stage I/II	155	0.1	24.9	0.8	1.4	0.5	199	0.3	12.1	0.9	1.4	0.7
	All cases	350	0.1	32.4	0.8	1.7	0.7	411	0.2	70	1	2.1	0.8
	benign condition							78	0.3	4.4	0.8	0.9	0.4
	healthy control	350	0.1	3.4	0.6	0.7	0.4	389	0.1	3.4	0.6	0.6	0.4

Supplementary Table 2: Sensitivity and specificity of MIC–1 in lung cancer patients

Specificity	Sensitivity (training set)				Sensitivity (validation set)			
	All cases Versus Healthy		Early stage Versus Healthy		All cases Versus healthy		Early stage Versus Healthy	
	Estimate	95% CI	Estimate	95% CI	Estimate	95% CI	Estimate	95% CI
0.99	0.440	38.7–49.4	0.381	30.4–46.2	0.397	34.9–44.6	0.362	29.5–43.3
0.97	0.709	87.7–93.9	0.665	58.4–73.8	0.672	62.4–71.7	0.628	55.7–69.5
0.95	0.849	80.7–88.4	0.794	72.1–85.4	0.844	80.6–87.8	0.839	78.1–88.7
0.90	0.911	87.7–93.9	0.890	83.0–93.5	0.891	85.6–91.9	0.879	82.6–92.1
0.80	0.963	93.7–98.0	0.942	89.3–97.3	0.939	91.2–96.0	0.930	88.5–96.1
0.70	0.974	95.2–98.8	0.961	91.8–98.6	0.966	94.4–98.1	0.975	94.2–99.2
0.60	0.983	96.3–99.4	0.968	92.6–98.9	0.973	95.3–98.7	0.985	95.7–99.7
0.50	0.994	98.0–99.9	0.994	96.5–100.0	0.985	96.8–99.5	0.990	96.4–99.9

Supplementary Table 3: Performances of MIC–1, Cyfra21–1, NSE, CA125, CEA and SCC for diagnosis of lung cancer

	Training set			Validation set		
	AUC (95% CI)	Sensitivity (%)	Specificity (%)	AUC (95% CI)	Sensitivity (%)	Specificity (%)
**all LC vs Healthy control**						
MIC–1	0.962 (0.945–0.975)	72.2	96.6	0.952 (0.935–0.966)	69.3	96.9
Cyfra21–1	0.847 (0.818–0.873)***	46.6	96.6	0.872 (0.847–0.895)***	48.7	96.9
NSE	0.806 (0.775–0.835)***	20.0	97.7	0.770 (0.739–0.798)***	18.7	99.2
CA125	0.787 (0.755–0.817)***	26.6	98.0	0.720 (0.688–0.751)***	25.8	97.9
CEA	0.756 (0.723–0.788)***	37.7	96.0	0.760 (0.729–0.789)***	36.3	95.9
SCC	0.682 (0.646–0.716)***	21.1	96.3	0.754 (0.723–0.784)***	26.3	96.6
**Early stage LC (stage I & II) vs Healthy control**						
MIC–1	0.953 (0.931–0.970)	67.1	96.6	0.953 (0.933–0.969)	65.3	96.9
Cyfra21–1	0.798 (0.760–0.832)***	34.2	96.6	0.808 (0.774–0.839)***	31.2	96.9
NSE	0.741 (0.700–0.779)***	6.5	97.7	0.677 (0.637–0.715)***	3.5	99.2
CA125	0.689 (0.646–0.729)***	5.8	98.0	0.616 (0.575–0.655)***	9.5	97.9
CEA	0.684 (0.642–0.725)***	20.6	96.0	0.688 (0.649–0.725)***	17.6	95.9
SCC	0.669 (0.626–0.709)***	15.5	96.3	0.739 (0.702–0.774)***	20.6	96.6
**Early stage LC (stage I & II) vs Benign condition**						
MIC–1				0.692 (0.634–0.746)	65.3	62.8
Cyfra21–1				0.724 (0.668–0.776)	31.2	97.4
NSE				0.525 (0.464–0.585)***	3.5	100
CA125				0.533 (0.473–0.594)**	9.5	96.2
CEA				0.665 (0.606–0.720)	17.6	93.6
SCC				0.601 (0.540–0.659)*	20.6	89.7

*$p < 0.05$, **$p < 0.01$, ***$p < 0.001$ in comparison to MIC–1.

Supplementary Table 4: ROC curve analysis in different histological subtypes

histological subtypes	Training group		Validation group	
	AUC	95% CI	AUC	95% CI
$NSCLC_{(scc)}$	0.965	0.945–0.980	0.949	0.927–0.967
$NSCLC_{(ca)}$	0.959	0.938–0.974	0.951	0.931–0.966
SCLC	0.968	0.945–0.983	0.958	0.934–0.975

scc: Squamous cell carcinomas; ca: denocarcinoma.

Supplementary Table 5: Multivariable logistic analyses for serum biomarkers and various diagnostic factors in patients with lung cancer

Variable	Odds Ratio	95% CI	*P*
Age, > 60 y vs ≤ 60	1.978	0.795–4.918	0.142
Sex, female vs male	1.669	0.855–3.261	0.134
MIC1	1.006	1.004–1.007	< 0.001
Cyfra21-1	1.902	1.493–2.422	< 0.001
CA125	1.034	1.010–1.059	0.006
CEA	1.129	0.996–1.280	0.058
NSE	1.066	1.000–1.136	0.051
SCC	1.107	0.620–1.977	0.732

Supplementary Table 6: AUC and sensitivities of different panels for diagnosis of lung cancer at 95% specificity in the training group

	All cases		Early-stage cases	
**Marker**	**AUC(95%CI)**	**sensitivity(95%CI)**	**AUC(95%CI)**	**sensitivity(95%CI)**
MIC–1	0.962 (0.945–0.975)	84.9% (80.7–88.4%)	0.953 (0.931–0.970)	79.4% (72.1–85.4%)
MIC–1+Cyfra21–1	0.974 (0.959–0.984)	86.9% (82.9–90.2%)	0.961 (0.941–0.976)	81.3% (74.2–87.1%)
MIC–1+Cyfra21–1+CA125	0.976 (0.961–0.986)	88.9% (85.1–92.0%)	0.963 (0.943–0.978)	82.6% (75.7–88.2%)
MIC–1+Cyfra21–1+NSE	0.975 (0.961–0.985)	87.7% (83.8–91.0%)	0.962 (0.942–0.977)	81.3% (74.2–87.1%)
MIC–1+Cyfra21–1+CEA	0.974 (0.960–0.985)	87.4% (83.5–90.7%)	0.961 (0.940–0.976)	81.3% (74.2–87.1%)
MIC–1+Cyfra21–1+CA125+NSE	0.977 (0.963–0.987)	88.0% (84.1–91.2%)	0.963 (0.943–0.978)	80.7% (73.5–86.5%)
MIC–1+Cyfra21–1+CA125+CEA	0.976 (0.962–0.986)	90.6% (87.0–93.4%)	0.962 (0.941–0.977)	83.9% (82.3–93.0%)
MIC–1+Cyfra21–1+CEA+NSE	0.976 (0.961–0.986)	88.6% (84.8–91.7%)	0.961 (0.941–0.976)	81.9% (75.0–87.6%)
MIC–1+Cyfra21–1+CA125+NSE+CEA	0.978 (0.959–0.984)	89.7% (86.0–92.7%)	0.963 (0.942–0.977)	82.6% (75.7–88.2%)

Supplementary Table 7: STARD checklist for reporting of studies of diagnostic accuracy. See Supplementary_Table_7
